# Supplementary figures and images for: Transmission of Klebsiella strains and plasmids within and between grey‐headed flying fox colonies
Source: Environ Microbiol. 2022 May 25;24(9):4425–36. doi: 10.1111/1462-2920.16047 (PMC9790207; doi:10.1111/1462-2920.16047)

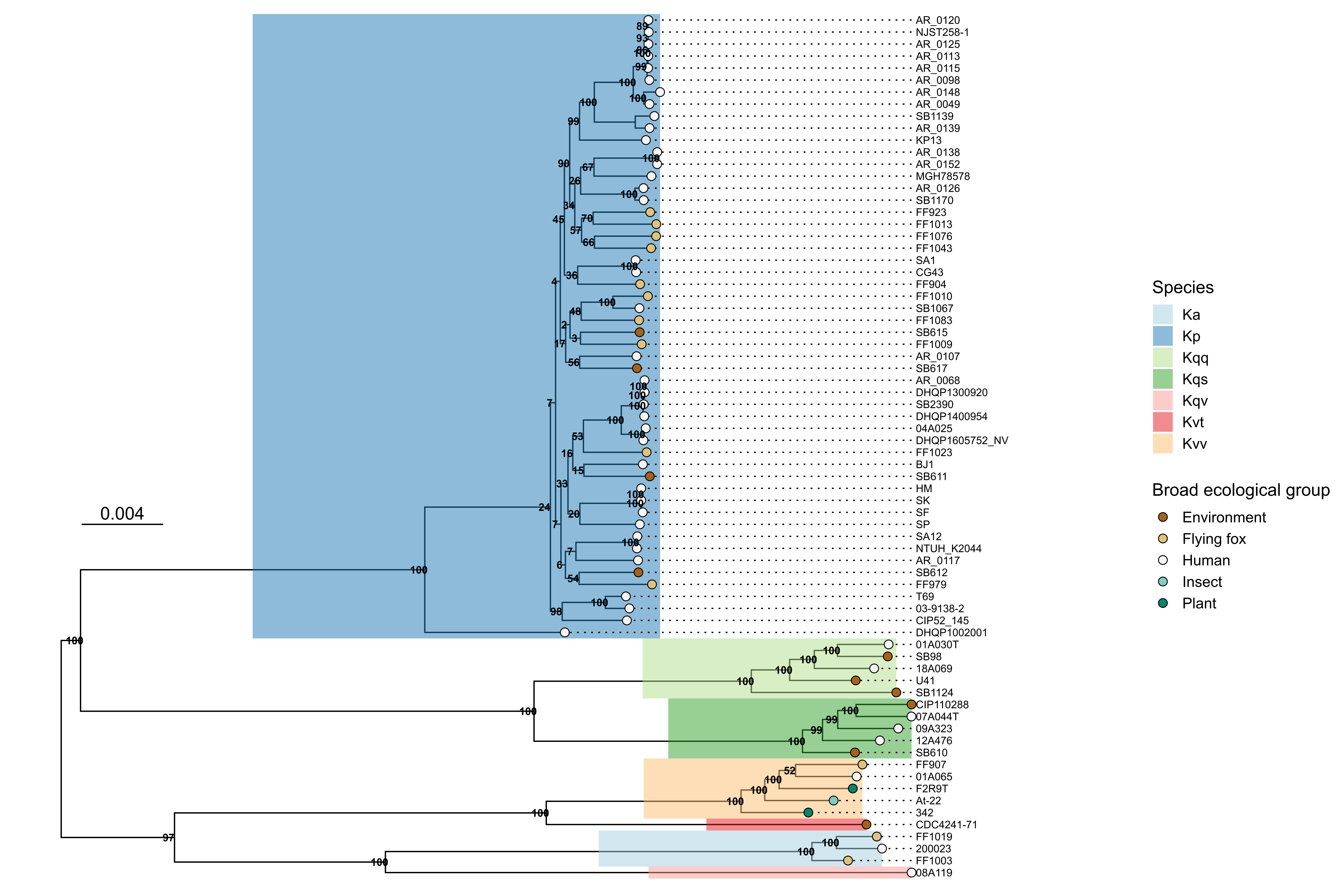

Supplement: Supplementary file 1 — Appendix S1. Supporting Information. [file EMI-24-4425-s001.zip › EMI_16047_Figure_S1.tif]

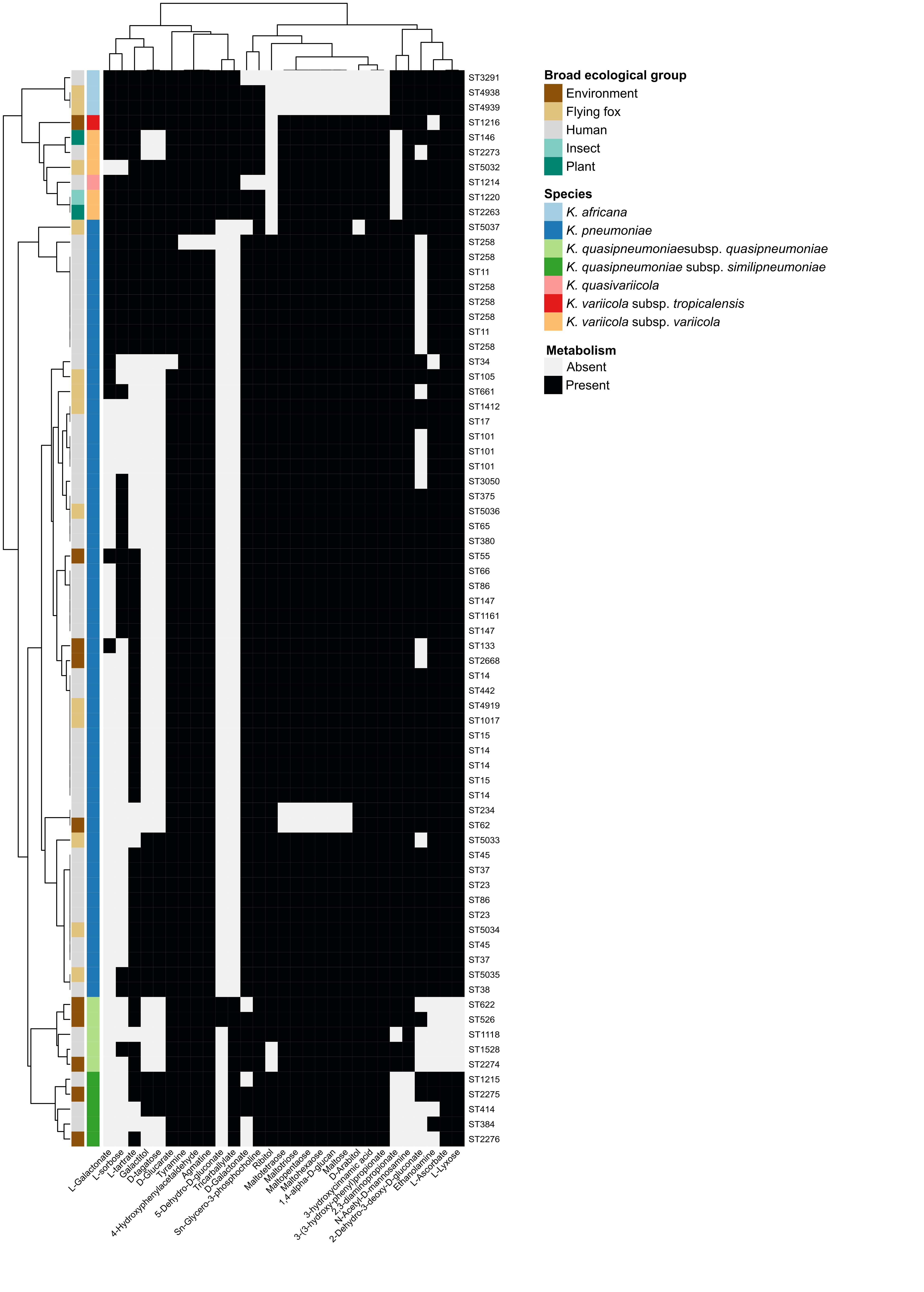

Supplement: Supplementary file 1 — Appendix S1. Supporting Information. [file EMI-24-4425-s001.zip › EMI_16047_Figure_S2.tif]

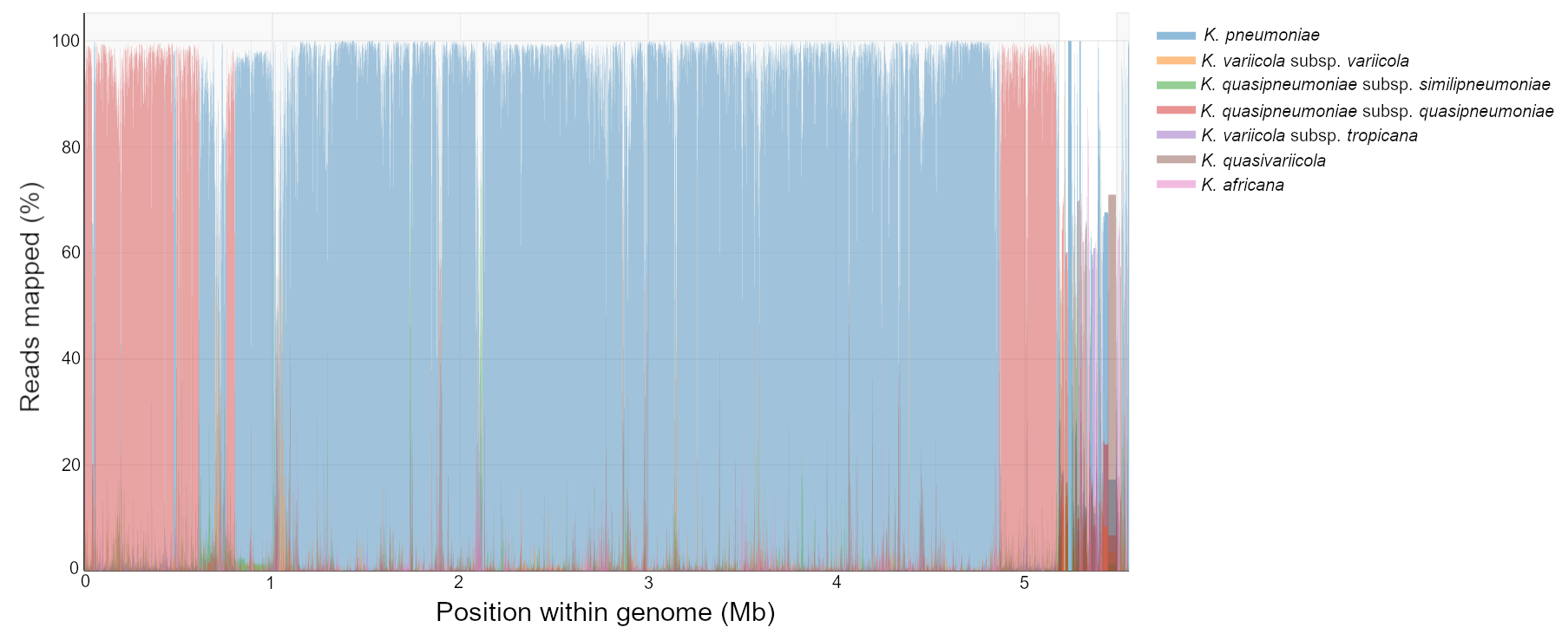

Supplement: Supplementary file 1 — Appendix S1. Supporting Information. [file EMI-24-4425-s001.zip › EMI_16047_Figure_S3.tif]
